# Force-dependent calcium signaling and its pathway of human neutrophils on P-selectin in flow

**DOI:** 10.1007/s13238-016-0364-4

**Published:** 2017-01-18

**Authors:** Bing Huang, Yingchen Ling, Jiangguo Lin, Xin Du, Ying Fang, Jianhua Wu

**Affiliations:** 1 School of Bioscience & Bioengineering, South China University of Technology, Guangzhou, 510006 China; 2 Hematology and Oncology Division, Guangdong General Hospital, Guangzhou, 510080 China

**Keywords:** neutrophils, P-selectin, calcium signaling, shear stress

## Abstract

P-selectin engagement of P-selectin glycoprotein ligand-1 (PSGL-1) causes circulating leukocytes to roll on and adhere to the vascular surface, and mediates intracellular calcium flux, a key but unclear event for subsequent arresting firmly at and migrating into the infection or injured tissue. Using a parallel plate flow chamber technique and intracellular calcium ion detector (Fluo-4 AM), the intracellular calcium flux of firmly adhered neutrophils on immobilized P-selectin in the absence of chemokines at various wall shear stresses was investigated here in real time by fluorescence microscopy. The results demonstrated that P-selectin engagement of PSGL-1 induced the intracellular calcium flux of firmly adhered neutrophils in flow, increasing P-selectin concentration enhanced cellular calcium signaling, and, force triggered, enhanced and quickened the cytoplasmic calcium bursting of neutrophils on immobilized P-selectin. This P-selectin-induced calcium signaling should come from intracellular calcium release rather than extracellular calcium influx, and be along the mechano-chemical signal pathway involving the cytoskeleton, moesin and Spleen tyrosine kinase (Syk). These results provide a novel insight into the mechano-chemical regulation mechanism for P-selectin-induced calcium signaling of neutrophils in flow.

## Introduction

Leukocyte activation is essential for innate immunity and resistance to pathogen infection and injury (Yuan et al., 2012), and cytoplasmic calcium concentration is a sensitive indicator of cell activation level during the trafficking of neutrophils into sites of inflammation (Schaff et al., 2008).

P-selectin, an adhesion molecule expressed on the surface of stimulated endothelial cells (Mcever, 2015), binds with P-selectin glycoprotein ligand-1 (PSGL-1) to mediate rolling and adhesion of circulating neutrophils (Ling et al., 2014; Mcever, 2015). Selectin engagement can trigger neutrophil calcium flux in the absence of chemokines under shear flow (Schaff et al., 2008; Zarbock and Ley, 2009). Increased cytoplasmic calcium concentration triggers cell activation and leads to superoxide generation (Romeo et al., 1975), enzyme secretion (Smolen et al., 1981), and actin gel-sol transitions (Yin and Stossel, 1979). The immune responses of leukocytes may be a mechano-chemical process. Force has been found to regulate cell rolling on selectins (Ling et al., 2014; Li et al., 2016), and to influence calcium signaling in T cells (Liu et al., 2014). High shear stress significantly enhanced calcium flux of neutrophils on E-selectin (Schaff et al., 2008), and calcium signaling in T-lymphocytes was also modulated by external force on the major histocompatibility complex (MHC)/T-cell receptor (TCR) complex (Liu et al., 2014).

The selectin-induced calcium signaling of neutrophils under flow might result from extracellular calcium influx or from intracellular calcium release (Clapham, 1995). Selectin engagement with PSGL-1 under tension might induce calcium influx directly from stretched calcium ion channels (Schaff et al., 2008). However, it was argued that binding of selectin to PSGL-1 could lead to activation of phospholipase C (PLC), and subsequently, the activated PLC would hydrolyze phosphatidylinositol 4, 5-bisphosphate (PIP2) to produce inositol-triphosphate (IP3), which subsequently mobilize calcium ion from non-mitochondrial stores (Rebecchi and Pentyala, 2000; Hogg et al., 2011; Stadtmann et al., 2011). In the pathway of P-selectin-induced calcium signaling, the initial event requires tyrosine phosphorylation of Syk through moesin, a member of the ezrin/radixin/moesin (ERM) family, which function as linkers of membrane protein (PSGL-1 and CD44)-actin cytoskeleton in neutrophils (Urzainqui et al., 2002; Matsumoto and Hirata, 2016). This further suggests that intracellular calcium release is cytoskeleton actin-dependent. The actin cytoskeleton is necessary for P-selectin-induced lymphocyte function-associated antigen 1 (LFA-1) activation, which also requires calcium bursting (Wang et al., 2006; Schaff et al., 2008). Lipid raft disruption alters the calcium signaling induced by G protein-coupled receptors (GPCR) and their ligands (Barabé et al., 2002), and FcγRIIIb triggers raft-dependent calcium influx in IgG-mediated responses in neutrophils (Marois et al., 2011). However, the role of lipid rafts on P-selectin-induced calcium signaling remains unclear.

Here, we demonstrated force-dependent intercellular calcium signaling of human neutrophils on immobilized P-selectin in the absence of chemokines under various shear stresses. The P-selectin-induced calcium signaling was detected using a parallel plate flow chamber (PPFC) and fluorescence microscope detection system, and scaled through the activation ratio, delay time, and the peak intensity of calcium flux (MATERIALS AND METHODS). Our data indicated that mechanical force triggered and regulated the calcium signaling of neutrophils firmly adhered on immobilized P-selectin in the absence of chemokines. Additionally, the calcium signaling was P-selectin concentration-dependent and resulted from intracellular calcium release rather than from extracellular calcium influx. Some molecules, such as cytoskeleton, moesin and Syk, were involved in the P-selectin-induced calcium signaling pathway. These results identify a mechano-chemical regulation mechanism for the P-selectin-induced calcium signaling of neutrophils under flow.

## Results

### Calcium bursting of neutrophils on immobilized P-selectin under flow

**Figure 1:**
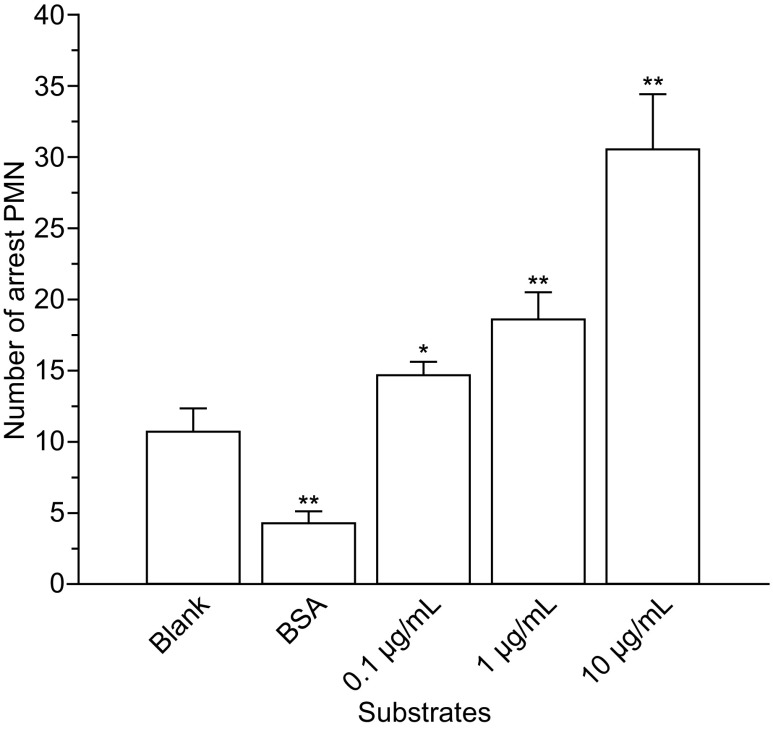
**Number of firmly adhered neutrophils on substrates with various treatments in flow**. The substrate was untreated, coated with BSA only, or coated with BSA or plus P-selectin (0.1, 1, or 10 μg/mL). The data represent the mean ± (standard error of mean, SEM) from 3 independent experiments at a wall shear stress of 0.2 Pa. The significant level of difference from the untreated substrate group is shown by *P*-value, * for *P* < 0.05 and ** for *P* < 0.01

To examine P-selectin-induced calcium signaling of circulating neutrophils under flow *in vitro*, we perfused 10^6^/mL neutrophil suspension over uncoated substrates or substrates coated with 1% bovine serum albumin (BSA) only, or BSA plus P-selectin (0.1, 1, or 10 μg/mL) under various wall shear stresses. We then counted the firm cell adhesive events and detected calcium bursting of the tethered neutrophils through fluorescence microscopy (MATERIALS AND METHODS). The firm adhesion events at a wall shear stress of 0.2 Pa occurred rarely for the substrates treated with nothing or with 1% BSA only compared to the number of events that occurred on substrates coated with 1% BSA supplemented with 0.1, 1, or 10 μg/mL P-selectin (Fig. 1), indicating that cell adhesion was specific for P-selectin.

**Figure 2:**
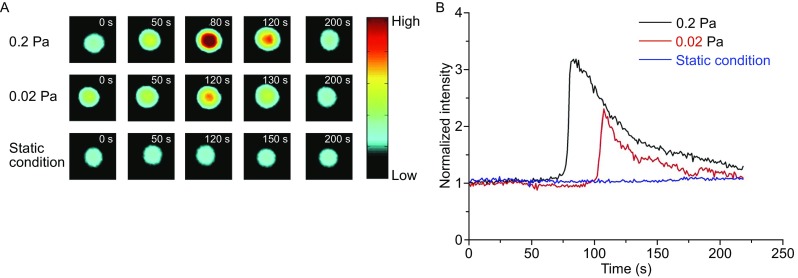
**Calcium bursting of firmly adhered neutrophils on P-selectin in flow**. (A) Three series of typical fluorescence images of firmly adhered neutrophils on immobilized P-selectin at different times, and (B) the time-course of the normalized fluorescence intensity of the cells over the observation time. Here, blue, red, and black represent the time courses performed with wall shear stresses of 0.0, 0.02, and 0.2 Pa on cells, respectively

The typical real-time dynamic process of calcium signaling of neutrophils on 10 μg/mL P-selectin-coated substrate in flow (Fig. 2A and 2B) exhibited a stable low level of cell fluorescence intensity of about one over the whole observation period of 200 s in the absence of wall shear stress, but under wall shear stresses of 0.02 or 0.2 Pa, the cell fluorescence intensity maintained constant only initially, then quickly increased to a peak and then gradually returned to its initial level. This indicated that calcium bursting of a firmly adhered cell required external force excitation, and occurred after a latent period or delay time (the time duration between the occurrence of firm cellular adhering at t = 0 and the sequent dramatic increase of the cell fluorescence intensity) of about 75 seconds for wall shear stress of 0.2 Pa or about 100 s for wall shear stress of 0.02 Pa (Fig. 2B). The peak calcium intensity, which was defined as the difference between the peak fluorescence intensity and the average of the normalized fluorescence intensity over delay time, indicates the maximum release of cytosolic calcium ions (Fig. 2B). This time-course of calcium signaling with a single peak (Fig. 2B) was similar to those described previously for neutrophils initially rolling on and then arresting at E-selectin in the presence of perfused chemokines (Schaff et al., 2008).

### P-selectin-induced calcium signaling of neutrophils was concentration-dependent

**Figure 3:**
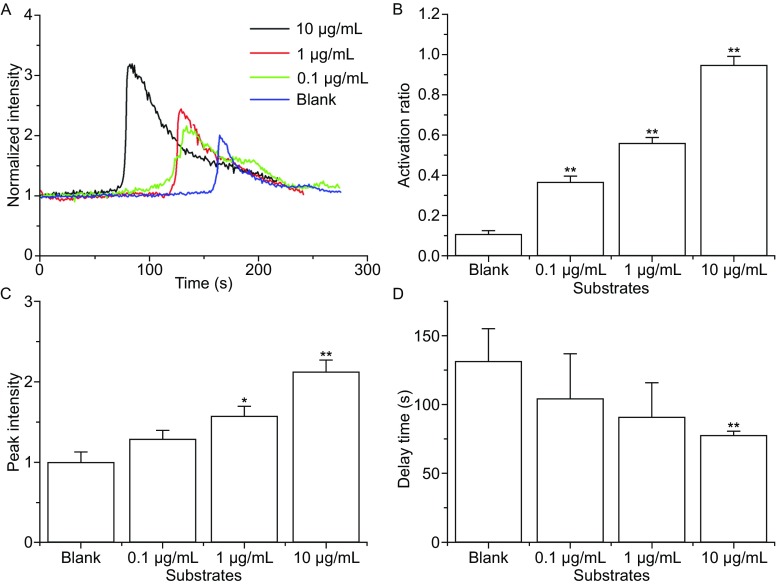
**Variation of activation ratio, peak intensity, and delay time for calcium signaling of firmly adhered neutrophils in flow versus P-selectin concentration**. (A) The time-course, (B) the activation ratio, (C) the peak intensity and (D) the delay time of calcium signaling with various P-selectin concentrations (0.0, 0.1, 1.0, and 10 μg/mL) at a wall shear stress of 0.2 Pa. The data represent the mean ± SEM from at least 20 cells in 3 independent experiments. The significant level of difference from the untreated substrate group is shown by *P*-value, * for *P* < 0.05 and ** for *P* < 0.01

To estimate the probability of cellular calcium signaling, we defined the cell activation ratio as the percentage of calcium signaling events in all firmly adhered cells over the 7 min observation period in the field of view (MATERIALS AND METHODS). In addition to adhesion, the calcium signaling of neutrophils should be also specific for the immobilized P-selectin, because that the activation ratio of neutrophils on the untreated substrate at a wall shear stress of 0.2 Pa was lower than that for the three treated substrates (Fig. 3B). The activation ratios of cells on substrates coated with P-selectin of 0.1, 1.0 and 10 μg/mL at a wall shear stress of 0.2 Pa were 36.5% ± 3%, 56% ± 3%, and 95% ± 4.3%, respectively (Fig. 3B), showing P-selectin concentration-enhanced calcium signaling of neutrophils.

The P-selectin concentration-enhanced calcium signaling was demonstrated also in the typical time-courses of calcium signaling with different treatments (Fig. 3A). The peak intensity of calcium signaling increased with P-selectin density (Fig. 3C), possibly duo to that increased interaction of P-selectin and PSGL-1 prompted cytosolic calcium release or extracellular calcium influx. The delay time of calcium signaling decreased with increasing P-selectin density (Fig. 3D), demonstrating that increasing interaction of P-selectin and PSGL-1 would increase the calcium signaling rate.

### Force triggered, enhanced, and quickened calcium signaling of neutrophils on immobilized P-selectin

**Figure 4:**
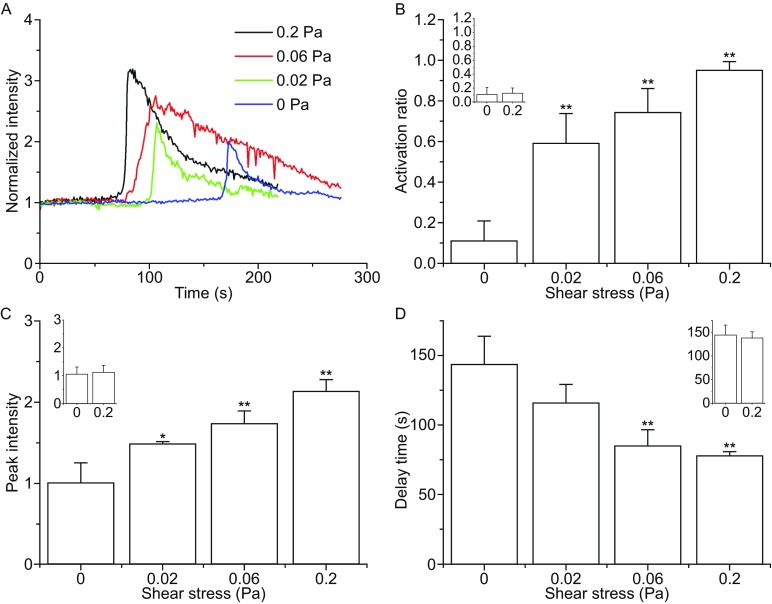
**Variation of activation ratio, peak intensity, and delay time for calcium signaling of firmly adhered neutrophils against shear stress**. The plots illustrate the time-course (A), activation ratio (B), peak intensity (C) and delay time (D) for calcium signaling of firmly adhered neutrophils on substrate coated with 10 μg/mL P-selectin under wall shear stresses of 0.0, 0.02, 0.06 and 0.2 Pa. The data represent the mean ± SEM from at least 20 cells in 3 experiments. Each inset in B, C or D presents the calcium signaling of neutrophils on blank substrate under wall shear stresses of zero and 0.2 Pa. The significant level of difference from static group was shown by *P*-value, * for *P* < 0.05 and ** for *P* < 0.01

External force was previously demonstrated to be necessary for calcium signaling that is induced by selectins (Schaff et al., 2008), but the mechanical regulation of P-selectin-induced calcium signaling of neutrophils remains unclear. Here, we examined the calcium signaling of neutrophils on substrate coated with 10 μg/mL P-selectin under wall shear stresses of 0.0, 0.02, 0.06, and 0.2 Pa. The typical time-courses of calcium signaling (Fig. 4A) showed that force regulated P-selectin-induced calcium bursting of neutrophils. The calcium bursting in the absence of stress was rare, weak, and slow, as shown by the very small activation ratio, low calcium peak, and long delay time in comparison with those under wall shear stresses 0.02 Pa (Fig. 4B–D). Increasing wall shear stress upregulated the cellular activation ratio (Fig. 4B), increased the peak level of calcium signaling (Fig. 4C), and shortened the delay time of calcium signaling (Fig. 4D). The insets in Fig. 4B–D illustrate that shear stresses did not alter the non-specific cellular calcium signaling. These results suggested that mechanical force served as a trigger and a positive regulator for P-selectin-induced calcium signaling of neutrophils. This phenomenon of force-enhanced calcium signaling may be relevant to the catch bond mechanism of interaction of P-selectin with PSGL-1, similar to the T-cell signaling triggered by the interaction of TCR to agonist peptide-MHC under external force (Liu et al., 2014). The catch bond should facilitate the signal transmission by stabilization of the engagement of P-selectin to PSGL-1 (Marshall et al., 2003; Ling et al., 2014).

### P-selectin induced calcium release rather than influx into neutrophils

**Figure 5:**
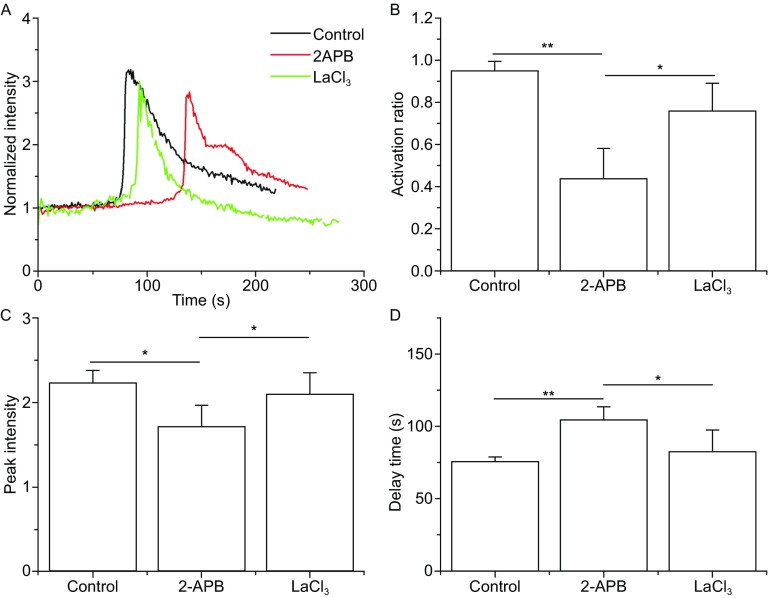
**Effects of blocking IP3 and membrane calcium channel on the calcium signaling of neutrophils on P-selectin in flow**. The time-course (A), activation ratio (B), peak intensity (C) and delay time (D) of P-selectin-induced calcium signaling of neutrophils, treated with the membrane calcium channel blocker LaCl_3_ and the IP3 inhibitor 2-APB or nothing, at a wall shear stress of 0.2 Pa. The data represent the mean ± SEM from at least 20 cells in 3 independent experiments. The significant level of difference from the control group (the cells treated with nothing) is shown by *P*-value, * *P* < 0.05, ** *P* < 0.01

To investigate whether the P-selectin-induced calcium bursting of neutrophils was due to cytosolic calcium release or extracellular calcium influx, we used 2-APB, an inositol trisphosphate receptor (IP3R) blocker, and LaCl_3,_ a plasma membrane calcium channel inhibitor. 2-APB or LaCl_3_-treated neutrophils (10^6^/mL) suspension were separately perfused over substrate coated with 10 μg/mL P-selectin at a wall shear stress of 0.2 Pa (MATERIALS AND METHODS). The typical time-courses of calcium signaling showed significantly differences for the different treatments (Fig. 5A). Treatment with 2-APB significantly reduced the activation ratio and peak intensity, and significantly increased the delay time (Fig. 5B–D). In other words, the IP3R blocker 2-APB could prevent, weaken and slow calcium bursting of neutrophils on P-selectin, suggesting that P-selectin induces human neutrophils calcium signaling by IP3 combining with IP3R, a main mediator in intracellular calcium release. Unlike the treatment with 2-APB, treatment with LaCl_3_ had no significant effects on cellular activation ratio, peak intensity, and delay time (Fig. 5B–D), demonstrating that blocking the calcium channel on cellular membrane with LaCl_3_ did not affect the calcium signaling of neutrophils. These results suggest that P-selectin induced human neutrophils calcium signaling through intracellular calcium release rather than through changes in extracellular calcium influx. However, the activation ratio and peak intensity remained considerable after blocking IP3R with 2-APB, suggesting that IP3 bound with IP3R might be only one of multiple mediators involved in intracellular calcium release

### Cytoskeleton actin and lipid raft were involved in P-selectin-induced calcium signaling of neutrophils in flow

**Figure 6:**
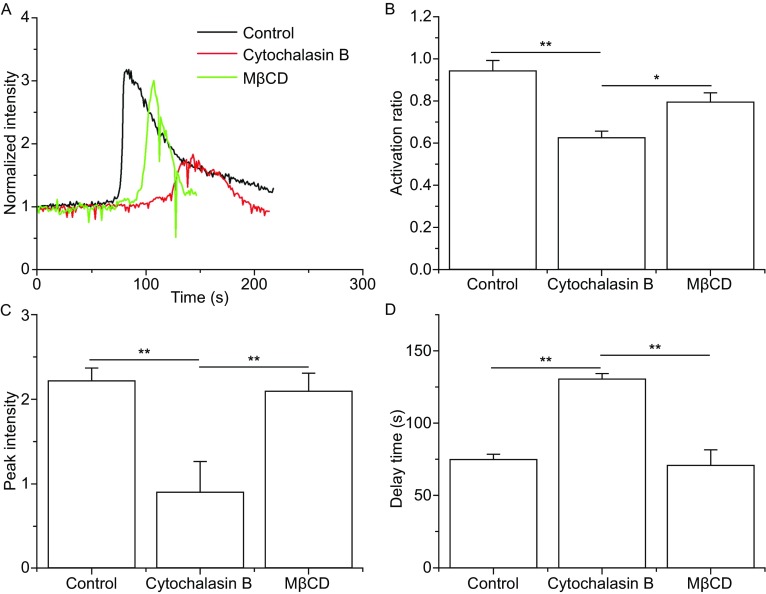
**Effects of cytoskeleton actin depolymerization and lipid raft disruption on calcium signaling of neutrophils on P-selectin in flow**. The time-course (A), activation ratio (B), peak intensity (C) and delay time (D) of P-selectin-induced calcium signaling of neutrophils, treated with or without cytochalasin B (cytoskeleton actin depolymerization agent) and MβCD (membrane lipid rafts disruption), at a wall shear stress of 0.2 Pa. The data represent the mean ± SEM from at least 20 cells in 3 independent experiments. The significant level of difference from the control group (the cells treated with nothing) is shown by *P*-value, * *P* < 0.05, ** *P* < 0.01

Both the cytoskeleton actin, the main intracellular mechanical mediator (Wang et al., 1993), and the lipid raft, the platform with many signal molecules (Horejsi and Hrdinka, 2014; Shao et al., 2015), are likely involved in the pathway of P-selectin-induced calcium signaling of human neutrophils. To test this involvement, we examined P-selectin-induced calcium bursting of neutrophils that were treated with the cytoskeleton actin inhibitor cytochalasin B (an actin depolymerization agent) or the lipid raft inhibitor methyl-β-cyclodextrin (MβCD) (MATERIALS AND METHODS) at wall shear stress of 0.2 Pa. The vehicle control experiment for cytochalasin B showed no significant difference between the vehicle and blank control (data not shown). The typical time-courses of calcium signaling showed significantly differences in different treatments (Fig. 6A). The cellular activation ratio was reduced from 0.95 to about 0.6 and 0.8 for treatments with cytochalasin B and MβCD, respectively, demonstrating a larger role for the cytoskeleton actin compared to the lipid raft in the P-selectin-induced calcium signaling of human neutrophils (Fig. 6B).

The treatment with cytochalasin B significantly reduced peak intensity and increased the delay-time, but the treatment with MβCD was indistinguishable from the control group (Fig. 6C and 6D), indicating that depolymerization of actin significantly weakens and slows calcium signaling, but the disruption of lipid raft only reduced occurring of calcium signal in neutrophils under flow. These data suggested that lipid rafts may stabilize both P-selectin engagement of PSGL-1 (Abbal et al., 2006; Yago et al., 2010) and act as a transmembrane transducer of calcium signaling (Kannan et al., 2007). Additionally, a stable conformation and rigid cytoskeleton are required not only for the formation of a transmembrane transducer but also for P-selectin-induced calcium signaling.

### P-selectin-induced calcium signaling of neutrophils requires moesin and Syk

**Figure 7:**
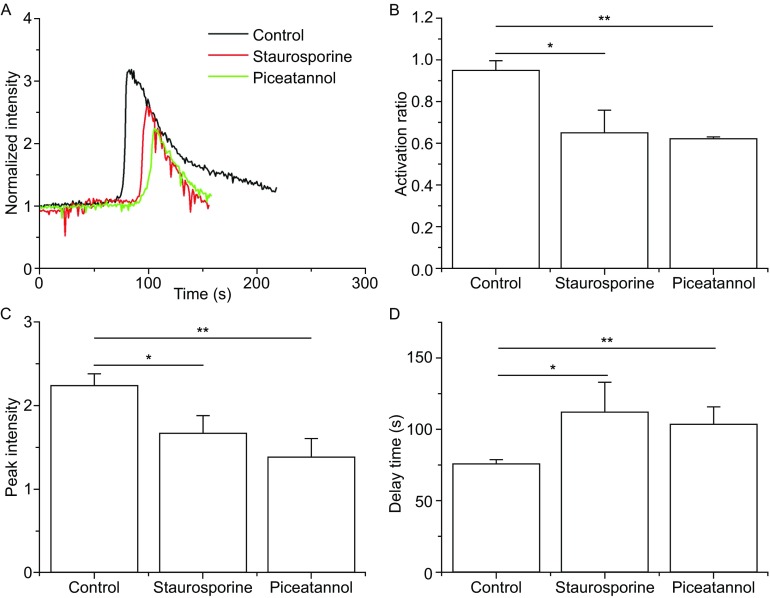
**Effects of blockage of moesin and Syk on P-selectin induce-calcium signaling in neutrophils in flow**. The time-course (A), activation ratio (B), peak intensity (C) and delay time (D) of P-selectin-induced calcium signaling of neutrophils, treated with or without moesin inhibitor staurosporine and Syk inhibitor piceatannol, at a wall shear stress of 0.2 Pa. The data represent the mean ± SEM from at least 20 cells in 3 independent experiments. The significant level of difference from control group (the cells treated with nothing) is shown by *P*-value, * *P* < 0.05, ** *P* < 0.01

Directly connected with actin and PSGL-1 (Serrador et al., 2002; Yago et al., 2010), moesin together with actin should be involved in the P-selectin-induced calcium signaling pathway. To test this hypothesis, the moesin inhibitor staurosporine was used to study the calcium signaling of neutrophils on P-selectin at a wall shear stress of 0.2 Pa (MATERIALS AND METHODS). The vehicle control experiment for staurosporine showed no significant difference between the vehicle and the blank control (data not shown). Figure 7A showed the typical time-course of calcium signaling under different conditions. We found that blocking moesin with staurosporine greatly reduced the activation radio and peak intensity, and lengthened the delay time in comparison with the control group (Fig. 7B–D), confirming the involvement of moesin in P-selectin-induced calcium signaling.

Syk is an important downstream signal molecular of moesin (Urzainqui et al., 2002; Matsumoto and Hirata, 2016) and can regulate calcium signaling in several immune cells (Kulathu et al., 2008; Krishnan et al., 2009). To determine if Syk mediates calcium signaling of human neutrophils on P-selectin, we measured calcium bursting of neutrophils at a wall shear stress of 0.2 Pa in the presence of the Syk inhibitor piceatannol (MATERIALS AND METHODS). The vehicle control experiment for piceatannol showed no significant difference between the vehicle and the blank control (data not shown). We found that blockage of Syk reduced the activation ratio and peak intensity dramatically, and significantly increased the delay time (Fig. 7B–D), suggesting that Syk is important for P-selectin-induced calcium signaling of neutrophils under flow.

## Discussion

During the trafficking of neutrophils into sites of inflammation, selectin supports the rolling and adhering of neutrophils by binding with PSGL-1 (McEver and Cummings, 1997). Selectin engagement with PSGL-1 under tension might induce force-dependent calcium flux (Schaff et al., 2008), through a pathway that includes tyrosine phosphorylation of Syk, activation of PLC, and subsequent hydrolyzation of PIP2 and production of IP3 (Rebecchi and Pentyala, 2000; Hogg et al., 2011; Stadtmann et al., 2011). However, the mechanical regulation of selectin-induced calcium signal transduction in human neutrophils was unclear. Here, we used a parallel plate flow chamber technique and fluorescent microscopy to study the calcium signaling of human neutrophils firmly adhered on immobilized P-selectin under various flow conditions. The results demonstrated that calcium signaling of neutrophils was induced by P-selectin, was triggered and enhanced by wall shear stress along a pathway that involved cytoskeleton actin, moesin and Syk.

In the previous studies of cellular calcium signaling such as of T cells under stretching force in the TCR-pMHC complex (Liu et al., 2014) and in neutrophils rolling on E-selectin and CD18 in the presence of chemokines (Schaff et al., 2008), the main focus was determination of the effect of external force on intensity. In contrast, we examined effects of shear stress not only on the intensity, but also on the delay time and the rate of calcium signaling in cells. This data showed that force could increase the rate and intensity of P-selectin-induced calcium signaling, but the addition of soluble P-selectin in suspension did not induce calcium signaling of neutrophils (data not shown). This finding suggested that the force-dependent calcium signaling of neutrophils on P-selectin required the shear stress-mediated buildup of tension on the P-selectin/PSGL-1 complex, as argued previously for the work on calcium flux of neutrophils rolling on E-selectin and CD18 (Schaff et al., 2008). Like the calcium signaling mediated by the TCR/MHC complex with force-regulated affinity on T cells (Liu et al., 2014), this force-dependent calcium signaling might be relevant to the “catch bond” mechanism that mediates the flow-enhanced roll and adhesion through a stabilizing interaction of selectin with PSGL-1 (Ling et al., 2014; Li et al., 2016). In this way, the P-selectin/PSGL-1 complex serves as a mechano-sensor to activate downstream intracellular signal molecules and to induce the calcium signaling of neutrophils under shear flow.

The P-selectin-induced calcium signaling should be Syk- and moesin-dependent (Fig. 7), because the calcium flux in neutrophils under flow was due to stored calcium release rather than extracellular calcium influx (Fig. 4). It was argued that the active immunoreceptor tyrosine-based activation motifs (ITAM) in moesin may trigger recruitment of Syk, and these activated Syk molecules could subsequently activate PLC (Hogg et al., 2011) to hydrolyze PIP2 into IP3, which would interact with its receptor on the endoplasmic reticulum (ER) to release calcium ions into the cytoplasm (Davies et al., 1990; Schaff et al., 2008; Falkenburger et al., 2013). This calcium signal pathway here was firstly demonstrated for P-selectin-induced calcium signaling, because blockage of both moesin and Syk caused significantly reduced calcium bursting of neutrophils on P-selectin in flow. This Syk-dependent calcium signaling also occurs in B cells and mast cells (Takata et al., 1994; Geahlen, 2014), and moesin is required for calcium signaling in response to TCR engagement in T cells (Shaffer et al., 2009).

Similar to ligated integrins (Müller et al., 2013), PSGL-1 bound to P-selectin should transmit a mechano-chemical signal through ezrin/radixin/moesin (ERM) proteins, which connect the cytoplasmic domain of PSGL-1 to the actin cytoskeleton (Snapp et al., 2002). The tension-induced rearrangement of actin cytoskeleton might mediate the clustering of lipid rafts (Yago et al., 2010) and further promote the P-selectin engagement of PSGL-1 and sequentially stabilize the downstream signaling. This argues that not only loosening the cytoskeleton but also disrupting rafts would reduce P-selectin-induced calcium signaling events in neutrophils under flow (Fig. 6B). Like loosening of the actin cytoskeleton, the blockage of moesin significantly reduced calcium signaling of neutrophils on P-selectin under flow (Figs. 6 and 7). The stretching of moesin may have activated ITAM, leading to the recruitment of Syk. Alternatively, the loosening of the actin cytoskeleton may not have promoted the stretching of moesin, hindering Syk activation and sequentially causing P-selectin-induced calcium signaling to become weak and slow (Figs. 6 and 7).

We here demonstrated that P-selectin engagement of PSGL-1 induced force-dependent calcium signaling of firmly adhered neutrophils in flow through a common pathway in which moesin, Syk, and cytoskeleton actin were required. The present results demonstrate a novel mechano-chemical regulation mechanism for the P-selectin-induced calcium signaling of firmly adhered neutrophils in flow and provide novel insight for our understanding of the inflammation response of leucocytes.

## Materials and methods

### Reagents

Recombinant Human P-Selectin/CD62P Fc Chimera Protein (R&D Systems, Minneapolis, MN) is a disulfide-linked homodimer containing the Fc moiety of human IgG and the extracellular domain of human P-selectin. Chemical IP3 inhibitor 2-APB, membrane calcium channel inhibitor LaCl_3_, Syk inhibitor piceatannol, lipid rafts disruption MβCD, cytoskeleton actin depolymerization agent cytochalasin B, and moesin inhibitor staurosporine were purchased from Sigma Chemical Co. (St Louis, MO, USA). All other reagents were of analytical grade or the best grade available.

### Isolation of human neutrophils

Neutrophils were collected from the whole blood of healthy volunteers according to the standard procedure, which was approved by the Research Ethics Committee, Guangdong General Hospital (Guangzhou, China).

First, 3 mL of Histopaque-1119 (Sigma Chemical Co., St. Louis, Mo.) was placed in a 15 mL conical centrifugation tube and 3 mL of Histopaque-1077 (Sigma Chemical Co., St. Louis, Mo.) was layered onto the Histopaque-1119. Fresh whole blood of 6 mL was anticoagulated with heparin, layered onto the density gradient, and then centrifuged at 700 g for 30 min at room temperature. Neutrophils were collected from the interface of Histopaque-1077 and Histopaque-1119 and into a new 15 mL conical centrifugation tube, and then washed with 10 mL phosphate buffer solution (PBS, Gibco, Grand Island, NY). After 10 min centrifugation at 400 ×*g*, the supernatant was discarded and 3 mL erythrocyte lysing buffer (ACK, Gibco, Grand Island, NY) was added into the tubes and gently shaken for 10 min. Then, the tubes were centrifuged for 10 min at 400 ×*g* and the supernatant was removed. The resulting neutrophils were re-suspended in PBS and assayed for cell viability using trypan blue exclusion. The obtained preparations were >95% pure and viable. All procedures were performed at room temperature.

### Flow chamber functionalization

The parallel-plate flow chamber (length × width × height = 2 cm × 0.25 cm × 0.0127 cm) was functionalized as described in our previous work (Ling et al., 2014; Li et al., 2016). Dry powder of P-selectin with Fc chain (R&D Systems, Minneapolis, MN, USA) was dissolved in PBS, and 20 µL of the solution was added into a coating region 2.5 mm × 5 mm of petri dish (Corning Glass Works, Corning, NY) bottom surface, which was held by a hollowed silicon gasket and marked in the cover slide center, and incubated overnight at 4°C. After removal of excessive unabsorbed P-selectin, the functionalization surface was washed with PBS containing 1% BSA (Calbiochem, San Diego, CA) 3 times, and incubated within the same solution for 1 h at room temperature to block nonspecific cells adhesion. The site densities of immobilized P-selectin on substrates were determined by the ^125^I radioiodination method (Ling et al., 2013). Mouse anti-human P-selectin mAB 9E1 (R&D system, MN) was labeled by the Pierce Iodination Kit (Thermo Fisher, IL) and purified by Sephadex G-25 column, then the ^125^I labeled antibody was added onto the P-selectin coated on the substrate. The radiation ^125^I intensity was detected by GE infinia Hawkeye 4 SPECT (GE Healthcare) after removing excessive antibody. The site densities of 0.1, 1.0, and 10 µg/mL P-selectin absorbed on polystyrene petri dish were determined to be 21, 208, and 1359 #/µm^2^ respectively. These P-selectin densities were selected to support the firm adhesion of neutrophils on the substrates.

### Loading with calcium sensitive dye and treating cells with inhibitors

The relative cytosolic calcium levels were estimated using the sensitive dye Fluo-4 acetoxymethyl (AM) ester (Invitrogen Life Technologies, Grand Island, NY, USA). 1 μmol/L Fluo-4 AM was loaded into neutrophils by incubating and gently vibrating cells (10^6^/mL) for 30 min at 37°C in loading buffer (20 mmol/L 4-(2-hydroxyethyl)-1-piperazineethanesulfonic acid (HEPES), 20 mmol/L glucose, and 1% BSA in PBS). After 10 min centrifugation at 400 ×*g*, neutrophils were re-suspended in loading buffer without dye, and, for complete de-esterification of intracellular AM ester, incubated for a further 30 min at 37°C until use.

To disrupt lipid rafts on membrane, depolymerize the cytoskeleton actin, or block IP3, the membrane calcium channel, Syk, and moesin, neutrophils were preincubated with lipid raft disruptor MβCD (5 mmol/L) and cytochalasin B (5 μg/mL) for 5 min, or IP3 inhibitor 2-APB (100 μmol/L) for 8 min, membrane calcium channel inhibitor LaCl_3_ (10 μmol/L) for 30 min, Syk inhibitor piceatannol (20 μmol/L) for 30 min, moesin inhibitor staurosporine (0.01 μmol/L) for 30 min, or 0.1% of DMSO as vehicle control.

### Cell adhesion and cytoplasm calcium assay in flow

The 10^6^ /mL labeled neutrophils were re-suspended in imaging buffer (110 mmol/L NaCl, 10 mmol/L KCl, 10 mmol/L glucose, 30 mmol/L HEPES, 1.5 mmol/L CaCl_2_, 1% BSA (*w*/*v*), and 12% Ficoll (*w*/*v*) at pH 7.35). With a syringe pump (Harvard PHD22/2000, Harvard Apparatus, Holliston, MA), the neutrophil suspension was perfused over the untreated, 1% BSA, or P-selectin (combined with 1% BSA)-coated bottom in a parallel-plate flow chamber at various wall shear stresses for 7 min (Ling et al., 2014; Li et al., 2016). A firm cell adhering event was defined as a cell movement with a distance less than 10 μm in 1 min. All adhesion events in the view window over the 7 min observation were counted. Images containing fluorescence of firmly adhered neutrophils were recorded by a QImaging Retiga-2000R digital camera coupled to an inverted microscope (Zeiss Axio Observer A1). Images were analyzed using Image Pro Plus v6.0 and Microsoft Excel 2010. The fluorescence intensity of a cell was normalized by FI_N_ = (FI_C_ – FI_B_) / FI_B_, where FI_N_ is the normalized cell fluorescence intensity, FI_C_ is the mean fluorescence intensity of the cell, and FI_B_, the fluorescence intensity of background, is the mean of four fluorescence intensities from four equidistant round domains (of 36π µm^2^) around the cell at a distance of 24 µm. The calcium signaling of neutrophils was characterized by three parameters, the activation ratio of the cell, the peak calcium intensity, and the delay time of cell calcium bursting. The cell activation ratio was defined as the percentage of calcium signaling events in all firmly adhered cells over the 7 min observation period under the field of view. The delay time of calcium bursting denoted the time duration between the occurrence of firm cellular adhering at t = 0 and the subsequent steep increase of cell fluorescence intensity. The peak calcium intensity was expressed by the difference between the peak fluorescence intensity and the average of the normalized fluorescence intensity over the delay time (Fig. 2A and 2B).

### Statistics methods

Student’s *t* test were used for the comparison of data. The data was considered significant if *P* values <0.05, and extremely significant if *P* values <0.01.
